# Detection of Neutralizing Antibodies against SARS-CoV-2 Post-Vaccination in Health Care Workers of a Large Tertiary Hospital in Spain by Using a Rapid Test LFIC and sVNT-ELISA

**DOI:** 10.3390/vaccines10040510

**Published:** 2022-03-25

**Authors:** José Tuells, Mónica Parra-Grande, Francisco J. Santos-Calle, Ana C. Montagud, Cecilia M. Egoavil, Celia García-Rivera, Pablo Caballero, Eva M. Gabaldón-Bravo, Juan Carlos Rodríguez-Diaz, José Antonio Hurtado-Sánchez

**Affiliations:** 1 Department Community Nursing, Preventive Medicine and History of Science, Alicante Institute for Health and Biomedical Research (ISABIAL), University of Alicante, 03690 Alicante, Spain; pablo.caballero@ua.es; 2 Microbiology Department, General University Hospital Alicante, 03010 Alicante, Spain; parra_mongra@gva.es (M.P.-G.); garcia_celriv@gva.es (C.G.-R.); rodriguez_juadia@gva.es (J.C.R.-D.); 3 Intensive Care Medicine Service, General University Hospital Elche, 03293 Elche, Spain; santos_fracal@gva.es; 4 Immunology Department, Fundación Jiménez Díaz University Hospital, 28040 Madrid, Spain; ana.cerda@quironsalud.es; 5 Clinical Pharmacology Unit, General University Hospital of Alicante, Alicante Institute for Health and Biomedical Research (ISABIAL), 03010 Alicante, Spain; egoavil_cec@gva.es; 6 Department of Nursing, Faculty of Health Sciences, University of Alicante, 03690 Alicante, Spain; eva.gabaldon@ua.es (E.M.G.-B.); ja.hurtado@ua.es (J.A.H.-S.)

**Keywords:** neutralizing antibodies, COVID-19, SARS-CoV-2, serological test, immunoassay, lateral flow assay, lateral flow immunochromatography, sensitivity and specificity

## Abstract

The presence of neutralizing antibodies (NAbs) against SARS-CoV-2 represent a surrogate marker of immunologic protection in populations at high risk of infection such as healthcare workers caring for hospitalized patients with COVID-19. As recommended by CDC and the European CDC, the use of rapid diagnostic tests during population-based evaluations offers an opportunity to identify individuals with serologic evidence of natural infection or who have undergone vaccination. We carried out a cross-sectional study to assess the presence of neutralizing antibodies against SARS-CoV-2 among medical providers at an intensive care unit of a large referral hospital in Alicante, Spain. In addition, we tested for the presence of neutralizing antibodies compared to serum of uninfected individuals from a Biobank. We were also interested in evaluating the use of a rapid lateral flow immunochromatography (LFIC) test against a surrogate ELISA viral neutralization test (sVNT). This rapid test demonstrated a specificity of 1.000 95% CI (0.91–1.00) and the sensitivity of 0.987 95% CI (0.93–1.00). The negative predictive value was 95%. After six months, this rapid test demonstrated that those immunized with two doses of BioNTech/Pfizer vaccine, maintained optimal levels of neutralizing antibodies. We concluded that all Health Care Workers develop NAbs and the use of this rapid immunochromatographic test represents a potential tool to be used in population-based studies to detect serological antibody responses to vaccination. Vaccination policies could benefit from this tool to assess additional doses of vaccine or boosters among high-risk populations.

## 1. Introduction

The pandemic caused by the SARS-CoV-2 coronavirus, the greatest health crisis in recent decades, is having a notable impact on the economy and a devastating effect in terms of health loss, causing millions of cases and deaths around the world [1,2]. During the initial waves of the pandemic, the highest case fatality occurred in the elderly [1,3]. However, front-line responders such as healthcare workers were also significantly affected [4] leading to many fatal cases [5] and a seroprevalence of around 5–9% [6,7]. Reduced availability of personal protective equipment during the initial part of the pandemic may explain the increased risk among healthcare workers.

Vaccines have been shown to be the best preventive tool to contain communicable diseases [8]. In the case of the emergence of SARS-CoV-2, the scientific community has obtained in record time different vaccines against this new disease, reaching a historic milestone in the field of vaccinology [2,8,9]. The mass vaccination campaigns deployed since December 2020 are reaching notable, although uneven progress. Different vaccination strategies are reducing the clinical severity and mortality from COVID-19, demonstrating the effectiveness of vaccines [10].

Immunity generated by vaccines is a broad process organized by a first phase of lymphocyte activation through the presentation of peptides from the vaccine protein by MHC molecules on the dendritic cell. For the next step, these activated T lymphocytes will assist in the activation of B lymphocytes involved in the process of production of specific antibodies to the vaccine protein. These phases are part of the mechanisms of cellular and humoral adaptive immunity that result in the generation of specific antibodies and immunological memory” [9,11,12]. During the last year and a half, numerous studies have been carried out to determine the degree of humoral immunity through studies of seroprevalence of COVID-19, both in the general population and in specific groups [3,13,14,15,16], useful for decision making.

Serological tests have been the most widely used tool to determine the immunological profile because they detect antibodies directed against structural proteins of the virus [13,17,18]. The Protein S or “Spike” is considered the entry route for the SARS-CoV-2 virus into the body through its binding to the ACE-2 receptor present in human cells [9,19]. For this reason, it has been the target of choice for the design of most commercialized vaccines [9,18,19,20]. Antibodies directed to the S1, S2 or RBD (“receptor binding domain”) regions of the S protein can block it and neutralize the entry of the virus into the cells. In fact, some studies have tested the neutralizing activity of these anti S1/S2/RBD antibodies against SARS-CoV-2 [21,22,23].

Health workers have been one of the priority groups to be vaccinated due to their exposure to the virus. The good data on vaccination coverage in this group have re-established a hospital safety environment reflected in the decrease in the in-hospital incidence [24,25]. Although there are studies that indicate durability of immunity [26,27,28], its long-term persistence remains unclear [18,20]. Recent studies in health workers immunized after infection revealed a duration of antibodies and a reduction in reinfection of at least 6 months [24,27]. However, the degree and potential of immunity achieved after vaccination against COVID-19 compared to natural immunity remains unclear [20].

In general, serological techniques work by detecting the presence of antibodies in biological samples based on high affinity binding to their specific antigen previously fixed on the corresponding technical platform [13,18]. The gold standard technical solution for detection antibodies is enzyme-linked immunofluorescense assay (ELISA). But conventional ELISA is not able to assess the neutralizing capacity of NAbs. For this, it is necessary to use cell cultures that allow it to be evaluated by measuring intracellular input. Unlike these gold standard viral neutralization tests, which require live cells and viruses, the new surrogate virus neutralization tests “surrogate Viral Neutralization Test” (sVNT) based on the “enzyme-linked immunofluorescense assay” (sVNT-ELISA) methodology has been developed [29,30]. The news tests have been shown to be effective in the detection of NAbs-SARS-CoV-2 [21,29]. This represents an important advantage, especially in its applicability in studies of seroprevalence and post-vaccination follow-up [30]. Recently, a rapid test for detecting NAbs-SARS-CoV-2 based on the principle of lateral flow immunochromatography (LFIC) has been commercialized. This test is cheaper, more manageable, and faster than sVNT-ELISA tests and cellular cultures. Hence, we believe that it will have greater applicability, both clinical and epidemiological studies.

The objective of our study was to compare the LFIC method against sVNT-ELISA tests, and to evaluate the prevalence of neutralizing antibodies generated by the exposure of a vaccine against SARS-CoV-2 after six months of complete regimen in high-risk Health Care Workers to the intensive care unit (ICU-HCW) at General University Hospital Elche (Spain).

## 2. Materials and Methods

### 2.1. Study Design and Participants

Observational cross-sectional study of a single centre carried out at the General University Hospital of Elche (Elche, Spain) on 14 July 2021. The study designed to independently validate the LFIC test consisted of a 2:1 unpaired case-control study.

The sample size for the prevalence study was calculated according to the efficacy estimated and reported by the BioNTech/Pfizer vaccine is >95% [31,32,33,34] determined by PCR. If an immune response with neutralizing antibodies occurs in a similar proportion [35,36] we perform the estimation of the sample with a confidence of 95%, and a precision of 5%, and assuming a 2% loss, which corresponds to 75 subjects.

The cases were voluntary ICU-HCW. The inclusion criterion in the study was established as working in the ICU in any job category and having received the complete schedule of the COVID-19 vaccine six months earlier. Exclusion criteria were the impossibility of providing informed consent to participate in the study. The controls were a set of samples stored in the ISABIAL Biobank from healthy patients or with non-infectious pathologies obtained during the pre-pandemic period, years 2015–2016.

### 2.2. Study Procedures

The full report of the procedure is detailed in the supplementary file. After distributing the information sheets to the participant and collecting the informed consents, serum samples were collected. All samples were stored at 4 °C for a maximum of seven days until processing. Regarding the control samples, the collection and processing conditions were SST tubes; centrifugation at 2000× *g* for 10 min at room temperature; aliquots of 500 μL frozen immediately at −80 °C. Prior to the analysis, samples were first brought to −20 °C and then fully thawed. The samples, both from the cases and from the controls, were coded and anonymized. Subsequently, all samples are analysed using ELISA techniques and a rapid LFIC test, by double-blinded members of the research team.

### 2.3. Serological Assays

The online Supplementary Material details information about the assays. All samples were evaluated simultaneously with the OJABIO^®^ SARS-CoV-2 Neutralizing Antibody Detection Kit (Colloidal Gold Method) of Wenzhou OJA Biotechnology Co., Ltd. (Wenzhou, China) technique that was based on a LFIC. The test was carried out according to the manufacturer’s recommendations. The result was interpreted manually by two trained investigators. The presence of visible bands was considered as positive.

The SARS-CoV-2 NeutraLISA assay from EUROIMMUN was used as a reference technique to validate the SARS-CoV-2 neutralizing antibody detection kit (OJABIO^®^ SARS-CoV-2 Neutralizing Antibody Detection). It is an ELISA test for the semi-quantitative in vitro determination of neutralizing antibodies against SARS-CoV-2 from serum or plasma. Both tests use the competitive inhibition of the protein-protein interaction between a recombinant SARS-CoV-2 S1/RBD protein and recombinant human ACE2 receptor to measure the specific neutralizing effect of antibodies in the patient sample. The test was carried out partially manually following the manufacturer’s instructions. The photometric measurement was carried out at a wavelength of 450 nm using the DS2^®^ Automated ELISA System. The results are reported in the form of percent iIDibition (% IH), by calculating a relationship between the extinction values of the controls or samples and the extinction value of the blank. Tests with a % IH ≥ 35 are considered positive and test with a % IH < 20 are considered negative.

### 2.4. Statistical Analysis

The sample size was estimated based on an expected sensitivity and specificity equal to that provided by the manufacturer, 94.8% and 99.2% respectively, a confidence level of 95% and a precision of 5% were considered. Finally, a case: control ratio of 2:1 was chosen. The variables collected for the cases were age, sex, history of COVID-19 infection, chronic pathologies, and recent respiratory infections (4 previous weeks), in addition to the brand of the vaccine received, the % IH and the result of the rapid test LFIC. For controls, only the result of the rapid test LFIC and % HI obtained from the sVNT-ELISA test were calculated. From these variables, % IH was calculated in the ICU-HCW according to the characteristics collected and the differences were analysed using the *t*-test and its equivalent in non-parametric the Mann–Whitney U test, always with a significance level of 0.05. In addition, to check if the % IH decreases over time or with age, the Pearson linear correlation coefficient was calculated between the time from the completion of the vaccination regimen to the date of serum collection and with declared age.

Regarding the verification of the LFIC rapid test, its sensitivity, specificity together with its 95% confidence intervals were calculated through the exact binomial and the curves of the positive and negative predictive value as a function of prevalence.

### 2.5. Ethical Considerations

The study was conducted in accordance with the principles from the Declaration of Helsinki related to human clinical trials, and the research proposal was approved by the Ethics Committee of the University of Alicante (Alicante, Spain) (File UA-2021-05-07_5, dated 24 May 2021), the General University Hospital of Elche (Elche, Spain) (File PI 59/2021, dated 22 June 2021), and the Department of Health, General University Hospital of Alicante (Spain) (File PI2021-094, Ref: 2021-0214, dated 30 June 2021).

## 3. Results

Current data from the Ministry of Health in the Valencian community region in Spain (2020) records that there were 43538 healthcare professional working in hospitals, including 8656 physicians (19.88%), 13705 nurses (31.48%), and 21177 additional staff members (48.64%). The study population characteristics in Table 1 show percentages similar to healthcare population workers in the Valencian Community [37,38,39]. Furthermore, the distribution by age and sex is like other hospitals in our region.

In total, 78 cases (volunteers) and 39 controls were analysed. All cases were vaccinated with two doses of mRNA COVID-19 (vaccines BioNTech/Pfizer), with a mean age of 42.76 years (95% CI 40.49–45.02), being 73.1% women, two of the cases had registered recent respiratory infections and 9 (11.4%) suffered from chronic diseases. The mean time between the 1st vaccination dose and the study was 181.3 days (6.0 months) and between the 2nd, 159.6 days (5.4 months), the standard deviation was 0.45 month in both cases. Characteristics of the volunteers are shown in Table 1.

Table 2 shows the percentage of inhibition (% IH) observed in the cases according to different characteristics. The correlation between % IH with age and time elapsed between the time of the study and the second dose was −0.046 (Sig. 0.687) and −0.201 (Sig. 0.077) respectively, without being statistically significant. The 12 individuals who had a history of SARS-CoV-2 infection were recovered together with their seroconversion after receiving the vaccine. Of the total, 71.8% had a previous PCR test and only 24.4% had done a previous serology test. These results show that the LFIC tests worked correctly without being influenced by age, gender, or previous history of COVID-19. The distribution of gender and age among HCW in this region is like that of other areas nationwide.

Table 3 lists the results of the verification of the new rapid test based on the results of the sVNT-ELISA test and its verification characteristics. No false-positive results were recorded, and the LFIC test registered a sensitivity of 98.7% and a specificity of 100%.

This table depicts the diagnostic accuracy of the assay, including specificity of 100% a positive predictive value of 100%. Given that vaccination coverage among healthcare workers is close to approximately 95%, the test’s specificity in this population will be relative to 100% (0.987). However, the specificity of this test will be lower in people with inadequate vaccination coverage.

**Figure 1:**
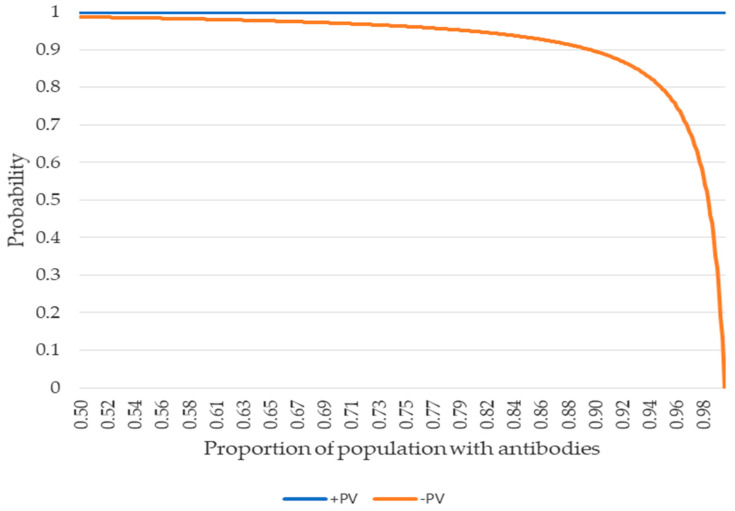
From the estimated sensitivity and specificity, the evolution of the positive (+PV) and negative (-PV) predictive values is shown for populations with a proportion of presence of antibodies greater than 50%.

Figure 1 shows the evolution of the predictive value for populations that exceed 50% of those vaccinated, as due to the high sensitivity and specificity of the test, in populations with less than 50%, the rapid test would be totally reliable. The negative predictive value knee is around 95%.

## 4. Discussion

There is a growing consensus for the use of neutralizing antibodies against SARS-CoV-2 as a tool for determining the degree of immunity and to achieve control of this pandemic. Thus, there is an urgent need to develop new and more accessible techniques for detecting these neutralizing antibodies. Epidemiological studies suggest the importance of achieving and adequate neutralizing antibody titer against SARS-CoV-2 after the infection has passed or after vaccination, to ensure a robust and prolonged immunity [13,20,24,28]. Taking this need into account, the two objectives of our study were to evaluate the efficiency of LFIC method for the detection of neutralizing antibodies and to evaluate the degree of humoral immunity achieved in a cohort of healthcare personnel six months after receiving the two doses of mRNA COVID-19 vaccines (BioNTech/Pfizer).

For the first objective, we obtained a high concordance (sensitivity and specificity) between the use of the LFIC rapid test and the sVNT-ELISA. Some authors have described the complexity of obtaining a consensus on the best strategy for accurate and high-throughput neutralizing antibody detection, as no test has been found to be performed with 100% sensitivity and specificity [13]. Dolscheid-Pommerich R et al. [21] and Rubio-Acero et al. [31] were the first to evaluate the concordance between the quantitative anti-S1 IgG levels determined by SARS-CoV-2 NeutraLISA assay from EUROIMMUN and the neutralizing antibody titers obtained by a microneutralization assay, obtaining in both cases a high correlation between both determinations. But so far, our study has been the only trial to verify the features of a rapid test by immunochromatography for the detection of neutralizing antibodies against SARS-CoV-2. Given that the available neutralization tests are not adequate to address routine large-scale tests, our results show a possibly cheaper, more accessible, and faster alternative (the test provides a result in 15 min compared to 3 h for the ELISA) to be able to assess the immune status and support epidemiological surveillance and vaccination programs.

Based on the predictive values calculated from the verification, the LFIC test could be very useful in populations with a proportion of people with antibodies less than 95%. This proportion is closely linked to the proportion of the vaccinated population. Therefore, it could be useful for the design of population vaccination strategies, such as the possibility of receiving boosters with second or third doses of vaccine, that is, to identify the moment to revaccinate populations with proportions of vaccinated less than 98% and 100%. This depends on the efficacy of the vaccine used in the population vaccination plans and the proportion of the population that does not generate antibodies when vaccinated, such as in the elderly (immunosenescence) or immunocompromised population.

In our second objective, it was observed how the % IH after six months remained with a mean value of around 85%. The presence of neutralizing antibodies against SARS-CoV-2 can serve as an indicator of the presence of protective immunity. But its duration is still unknown, and the necessary threshold level of neutralizing antibodies has not been established. According to the data published in the literature, more than 90% of the subjects generate neutralizing antibodies, which remain elevated and stable after the first 5–6 months of infection/vaccination [20,26,27,28], data that agree with those obtained in our study. Nevertheless, Israel et al. reported a gradual increase in the risk of infection following the second vaccine dose after at least 90 days with a risk of infection after six months of 2.82%, supporting a booster dose at 3–4 months after administration second dose [40]. Although we have not found evidence of a decrease in humoral immunity six months after receiving the two doses of vaccine, the trend of % IH should continue to be monitored, especially to decide which population and when for planning of a new doses.

On the other hand, we did not find significant differences in % IH based on age, sex, and history of COVID-19 infection. These data are consistent with data published in the study by Ripperger et al. [26] where, contrary to expectations, they did not observe differences in the humoral response depending on the age and sex of the subjects, although it may be due to sampling and representation.

It is noteworthy that the only negative case with the LFIC test and with a positive sVNT-ELISA test, but with a lower limit of antibodies, turned out to be an immunosuppressed person under treatment with a biological drug to control an autoimmune disease. This reinforces the thesis of the possible need for revaccination in these groups [41].

Our study has some important limitations including potential biases such as the lack of definitive gold standard test, and the range of the sample was broader to include populations to which the test will not be administered in clinical practice and in this manner increase the validity of the test. In our study, we were able to control for history of vaccination (by reviewing their vaccination certificate), or not receiving vaccination (since controls were serum samples obtained from a biobank of sera from individuals prior to the pandemic. The concomitant use of ELISA to confirm or not history of vaccination increases the validity of the assay.

Finally, the OJABIO test was done independently of the ELISA test, on different days and by different laboratory personnel. The tests were included during routine activities of laboratory technicians, guaranteeing independence and blindness from the study. The sample of confirmed COVID-19 cases includes populations of individuals with chronic diseases (11.3%), persons who recovered from COVID-19 (15.4%), and vaccinated individuals between 2.2 and 5.2 months. Therefore, the spectrum of the sample was wide enough to ensure the diagnostic validity of the rapid test

One of the limitations of the study was that all the participants had received the Pfizer/BioNTech vaccine with a complete regimen (two doses), so the response to other vaccines has not been proven and this regimen could justify the extremely high values for most samples in the sVNT-ELISA essay [42]. Regarding the limitations of verification, the design allowed us to avoid verification and incorporation biases, concerning spectrum bias, the cases included 11% of people with different chronic pathologies, so this possible bias is controlled to a certain extent. In addition, the second control group missing here, that of natural infections because it is difficult to collect active cases, as almost 80% of the Spanish population is currently vaccinated.

The emergence of new variants of SARS-CoV-2 after this study was conducted including Omicron variant with the ability to evade neutralizing antibodies, is a limitation of this study. We are planning to conduct an evaluation of the utility of this test with Omicron infections [43,44]. Indeed, epidemiological follow-up of the immune response to vaccination depending on the evolution of the viral variants is an important best public health practice.

Finally, we can assume the neutralizing character of these antibodies by the sVNT-ELISA technique together with a correlation of the results allows us to suggest the detection of post-vaccine neutralizing antibodies (protein used in study vaccines). Although this ability is indeed guaranteed by the sVNT-ELISA technique and by the literature published about anti-Spike protein antibodies [21,22,23]. We do not believe it necessary to make a comparison with techniques that include cell cultures and viruses, neither the complexity that it implies, nor the use that LFIC has as a screening method and epidemiological tool in the research framework. However, further studies are required to assess the cut-off, considering different populations and variants of COVID-19.

## 5. Conclusions

In conclusion, we could affirm that HCW immunized with two doses of mRNA COVID-19 vaccines (BioNTech/Pfizer) maintain a good level of NAbs six months after being vaccinated. Therefore, the LFIC test can be one more tool in immunization campaigns and programs evaluation. From a public health perspective, they can be helpful in studies in epidemiological surveillance systems or for decision-making in vaccination strategies and follow-up of the immune response to vaccination in a population to cope with new variants. Likewise, the pandemic crisis has shown the need to vaccinate globally to mitigate it. However, places like the African continent offer low vaccination coverage compared to rich countries that have already implemented the third vaccine. These rapid tests could save on vaccine doses allowing them to be sent to low-income countries that need them.

## Figures and Tables

**Table 1:** Socio-demographic and clinicopathological characteristics of the subjects included in this study.

Variable	*n*	%	95% CI
**Age**			
<40 years	29	37.2	(26.5–47.9)
≥40 years	49	62.8	(52.1–73.5)
**Gender**			
Male	21	26.9	(17.1–36.8)
Female	57	73.1	(63.2–82.9)
**Occupation Activity**			
Nurses	36	46.2	(35.1–57.2)
Doctors	16	20.5	(11.6–29.5)
Others	26	33.3	(22.9–43.8)
**History of infection COVID-19**			
Yes	12	15.4	(7.4–23.4)
No	66	84.6	(76.6–92.6)
**Previous PCR**			
Yes	56	71.8	(61.8–81.8)
No	22	28.2	(18.2–38.2)
**Previous Serology**			
Yes	19	24.4	(14.8–33.9)
No	59	75.6	(66.1–85.2)
**Baseline chronic disease**			
Yes	9	11.3	(4.4–18.6)
No	69	88.5	(81.4–95.6)
Recent Respiratory Infection (4 weeks) *			
Yes	2	2.6	(0.0–6.1)
No	76	97.4	(93.9–100)

* ageusia, or at least three symptoms among fever; chills; severe tiredness; sore throat; cough; shortness of breath; headache; or nausea, vomiting, or diarrhoea.

**Table 2:** Percentage of inhibition (% IH) according to age, sex, and history of COVID-19 infection in the group of cases.

Variable	*n* (%)	% IH Mean	% IM SD	95% CI	Sig.	U-Sig.
Total Cases	79 (100%)	84.55	12.64	(84.70–87.40)	**-**	79 (100%)
Age						
Older or same 40	49 (62.8)	83.80	13.14	(80.03–87.58)	NS	NS
Younger than 40	29 (37.2)	85.82	11.86	(81.32–90.33)		
Gender						
Female	57 (73.1)	84.29	13.41	(80.73–87.85)	NS	NS
Male	21 (26.9)	85.26	10.54	(80.47–90.06)		
COVID-19						
Yes	12 (15.4)	88.57	13.41	(80.04–97.09)	NS	NS
No	66 (84.6)	83.82	12.47	(80.76–86.89)		

SD: Standard Deviation, 95% CI: 95% confidence interval, Sig.: Statistical significance *t*-test. U-Sig.: Statistical significance U Mann–Whitney test. NS: No statistically significant.

**Table 3:** Sensitivity and specificity, with 95% confidence intervals (CIs), for LFIC test.

		Results sVNT-ELISA		Total
		Positive	Negative	
**Results LFIC**	Positive	77	0	77
	Negative	1	39	40
	Total	78	39	117
Test characteristics				
Sensitivity	0.987 95% CI (0.93–1.00)			
False Negative	0.013 95% CI (0.00–0.07)			
Specificity	1.000 95% CI (0.91–1.00)			
False Positive	0.000 95% CI (0.00–0.09)			
False Positive	0.000 95% CI (0.00–0.09)			

95% CI. 95% Confidence interval for an exact binomial test. Lateral flow immunochromatog-raphy: (LFIC); surrogate Viral Neutralization Test (sVNT).

## Data Availability

Not applicable.

## Supplementary Materials

The following supporting information can be downloaded at: https://www.mdpi.com/article/10.3390/vaccines10040510/s1: Validation Report "Detection of Neutralizing Antibodies against SARS-CoV-2 (COVID-19)", General University Hospital Alicante (Alicante, Spain).
