# Supplementary material for: Detection of Neutralizing Antibodies against SARS-CoV-2 Post-Vaccination in Health Care Workers of a Large Tertiary Hospital in Spain by Using a Rapid Test LFIC and sVNT-ELISA
Source: Vaccines (Basel). 2022 Mar 25;10(4):510. doi: 10.3390/vaccines10040510 (PMC9032347; doi:10.3390/vaccines10040510)
Supplement: Supplementary file 1 [file vaccines-10-00510-s001.zip › vaccines-1597806-supplementary.pdf]

VALIDATION REPORT

OJABIO RAPID TEST

Detection of Neutralizing Antibodies against SARS-CoV-2 (Covid-19)

REPORT CONTENT

1. OBJECT AND FIELD OF APPLICATION 3

2. APPLICABLE PROCEDURES AND DOCUMENTS 3

3. TECHNICAL AND HUMAN RESOURCES 4

4. MATERIALS AND EQUIPMENT NEEDED 4

5. GENERAL DESCRIPTION OF THE KIT TO BE VALIDATED AND REFERENCE ELISA 5

6. SAMPLES USED IN THE STUDY 7

7. VALIDATION PROTOCOL 8

7.1. Operating process 8

7.2. Validation parameters 8

8. VALIDATION RESULTS 9

9. VALIDATION CONCLUSIONS 11

1. PURPOSE AND FIELD OF APPLICATION

The purpose of this report is to carry out the necessary tests to demonstrate the technical validity of the Rapid Test of Neutralization Antibodies (AcN) of SARS-CoV-2 of the commercial house of OJABIO (hereinafter Rapid Test AcN SARS-CoV-2 of OJABIO), through its comparison with the reference method (NeutraLISA SARS-CoV2, Euroimmun)

The OJABIO AcN SARS-CoV-2 Rapid Test is a qualitative serological test used for the detection of neutralizing antibodies against coronavirus in direct blood and / or serum samples.

Validation is performed by comparison with the results obtained through the reference method (NeutraLISA SARS-CoV2, Euroimmun), with respect to the results obtained with the OJABIO AcN SARS-CoV-2 Rapid Test.

The necessary tests for the validation of the test are carried out by the Microbiology Service of the General University Hospital of Alicante. The Service is accredited according to the ISO 9001: 2015 standard (Certificate number ES117080-1)

Wenzhou OJA Biotechnology CO. LTD (China), authorized distributor of the OJABIO AcN SARS-CoV-2 Rapid Test in Spain, provides the Service with 6 kits for validation, on 06/01/2021.

For the sensitivity and specificity results obtained after validation, it is considered that the OJABIO AcN SARS-CoV-2 Rapid Test is suitable for the qualitative detection of neutralizing antibodies against coronavirus under the conditions evaluated.

The results of the validation are authorized by Dr. Juan Carlos Rodríguez Díaz, Head of the Microbiology Section of the General University Hospital of Alicante.

2. APPLICABLE PROCEDURES AND DOCUMENTS

For the validation of the kit, the following documents, among others, have been taken as a reference:

- Standard UNE-EN ISO 15189: 2013 Clinical Laboratories. General requirements for quality and competence.

- CEA-ENAC-20 Rev. 1 Specific Accreditation Criteria. Microbiological analysis, ENAC May / 2017.

- G-ENAC-20 Rev. 1 Guide for the selection and use of test kits by accredited laboratories, ENAC / 2017.

- Validation and analytical verification of microbiological methods, SEIMC 2013.

- Instructions for use and processing of the OJABIO AcN SARS-CoV-2 Rapid Test.

- Instructions for use and processing of the Euroimmun NeutraLISA SARS-CoV2 assay.

- CE Declaration of Conformity OJABIO AcN SARS-CoV-2 Rapid Test, September / 2020.

- Approval of the Ethics Committee of the University of Alicante (Spain) (File UA-2021-05-07_5, dated 05/24/2021).

- Approval of the Ethics Committee for Drug Research of the General University Hospital of Elche (Spain) (File PI 59/2021, dated 06/22/2021).

- Approval of the Ethical Committee for Research with Medicines of the Alicante Department of Health, Hospital General (Spain) (File PI2021-094, Ref .: 2021-0214, dated 06/30/2021).

3. TECHNICAL AND HUMAN RESOURCES

The Microbiology Service has an integrated quality management system, under ISO 9001: 2015 standards. This integrated system allows the service to be managed globally, including processes such as:

- Standardization of analytical processes, through the availability of standard work procedures (SOPs) for each of them.
- Traceability of processes.
- Management and control of equipment maintenance, verification and calibration activities.
- Planning of internal and external quality control activities.

4. MATERIALS AND EQUIPMENT NEEDED

- OJABIO SARS-CoV-2 AcN Rapid Test Kit

- Euroimmun SARS-CoV2 NeutraLISA Kit

Additional Materials and Equipment (not included in the Test Kit):

- ELISA DS2® automated system
- Calibrated Pipettes and Pipette Tips
- 1.5mL eppendor tubes
- Distilled or deionized water
- Incubator for incubation at + 37 ° C

5. GENERAL DESCRIPTION OF THE KIT TO BE VALIDATED AND THE REFERENCE ELISA

5.1. OJABIO SARS - CoV - 2 Neutralization Antibody Rapid Test

OJABIO Rapid test Detection Kit for Neutralizing Antibodies against SARS-CoV-2 is a lateral flow immunoassay for the qualitative detection of neutralizing antibodies against SARS-CoV-2 in serum, plasma, venous or peripheral whole blood samples. It is intended to be used as a screening test, and helps to determine previous exposure to the virus, to verify the efficacy of vaccines against SARS-CoV-2, and for epidemiological purposes.

The OJABIO AcN SARS-CoV-2 Rapid Test is based on a lateral flow immunochromatography for the qualitative determination of neutralizing antibodies to SARS-CoV-2. Neutralizing antibodies are immunoglobulins that target the receptor binding domain (S-RBD) of the S protein or spike of the SARS-CoV-2 type coronavirus, they can be of the IgM, IgG and / or IgA isotype. Neutralizing antibodies are detected by the specific recognition of neutralizing antibodies by the recombinant S-RBD antigen of SARS-CoV-2. The T line of the SARS-CoV-2 neutralizing antibody test card was coated with the recombinant SARS-CoV-2 antigen S-RBD, and the C line was coated with multiple sheep anti-S-RBD antibodies.

By dispensing the sample and diluent into the indicated well, the sample migrates by capillary action through the cassette. If there are neutralizing antibodies to SARS-CoV-2, they will bind to the colloidal gold particles immobilized in the matrix prior to the reaction matrix, forming an analyte (AcN) -colloidal gold * conjugate. Next, in the reaction matrix (line T) is the recombinant S-RBD antigen of SARS-CoV-2 fixed to the nitrocellulose membrane, if the AcN * are present they will bind specifically to the antigen forming an antigen complex -antibody. The presence of these immune complexes will be revealed by the appearance of a reddish band.

The test contains an internal control (C band) that should exhibit a reddish band regardless of color development in the T band. Otherwise, the test result is invalid and the sample must be retested with another device.

Interpretation of results:

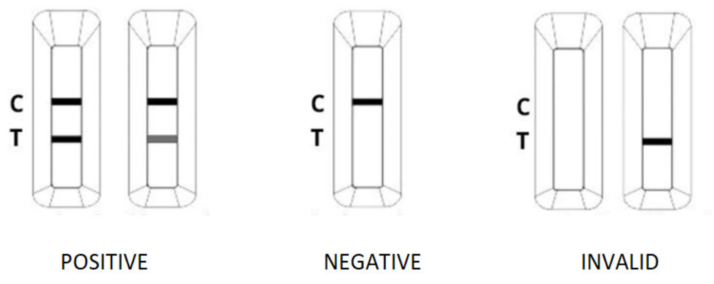

5.2. Euroimmun NeutraLISA SARS-CoV2 Assay

The Euroimmun NeutraLISA SARS-CoV2 Assay is an enzyme-linked immunosorbent assay (ELISA) for the semi-quantitative in vitro determination of neutralizing antibodies against SARS-CoV-2 for serum or plasma. The assay contains microplate strips with 8 reagent wells, coated with S1 / RBD domains of SARS-CoV-2 protein S. In the first step of the analysis, samples diluted with a buffer containing ACE2 are incubated in the reagent wells. If the sample contains neutralizing antibodies, they compete with the ACE2 receptor for the binding sites of the RBD / S1 protein. Unbound ACE2 is removed by a subsequent wash step. For the detection of bound ACE2, in a second step it is incubated with peroxidase-labeled streptavidin which, in a third step, catalyzes the colored reaction. The test was carried out partially by hand following the manufacturer's instructions. The photometric measurement was performed at a wavelength of 450 nm using the DS2® Automated ELISA System. The results are reported in the form of percent inhibition (% HI), by calculating a relationship between the extinction values of the controls or samples and the extinction value of the blank. Those values with a % HI ≥ 35 being positive and negative values with a % HI <20.

Technical data:

Antigens

Solid phase: S1 / RBD domain of SARS-CoV-2 protein S

Liquid phase: biotinylated human ACE2 receptor (angiotensin converting enzyme 2).

Test evaluation

Semi-quantitative, the percentage of inhibition (% HI) is calculated. The intensity of the absorbance is inversely proportional to the concentration of neutralizing antibodies in the sample.

% HI = 100% - (sample absorbance x 100% / blank absorbance).

Cut-off point at 25% HI (borderline 20-35% HI)

Interpretation

% HI <20: negative

% HI ≥ 20 - <35: borderline

% HI ≥ 35: positive

Sample dilution

Serum or plasma, 1: 5 in sample buffer previously prepared with ACE2.

Test procedure: 60 min (37 ° C) / 30 min (RT) / 15 min (RT) (sample / conjugate / substrate). Automatable.

Kit format: 96 divisible wells.

Reference: EI 2606-9601-4, SARS-CoV-2 NeutraLisa CE

6. SAMPLES USED IN THE STUDY

For the validation, 78 serum samples were selected from 78 health workers from the Intensive Care Unit (ICU) of the General University Hospital of Elche vaccinated with the two doses of the Pfizer-BioNTech vaccine and who presented a serological result prior to vaccination negative for SARS-CoV2. The negative controls (for every two participants, a negative control was included), frozen plasma was selected, guarded by the Biobank of the General Hospital of Alicante - ISABIAL, from 39 subjects who have donated as healthy controls or with stage I oncological process without treatment oncology at the time of donation to the Biobank and that were collected in the 2015-2016 period prior to the pandemic.

7. VALIDATION PROTOCOL

7.1. Operating process

Validation is performed by comparison between the results obtained through the ELISA reference method, with respect to the results obtained with the OJABIO AcN SARS-CoV-2 Rapid Test.

For validation, all samples are processed by ELISA, and in parallel by the AcN SARS-CoV-2 Rapid Test from OJABIO. After completing the operational process, the results obtained are compared.

The ELISA methodology allows to quantify the concentration of neutralizing antibodies. For the equipment used in the validation, the cutoff values are indicated below:

% Percentage Inhibition	Interpretation
**<20**	Negative
**20-35**	Indeterminate
**≥35**	Positive

7.2. Validation parameters

From the results obtained in the validation, the following validation parameters will be calculated:

- **Analytical sensitivity:** proportion of samples that contain the investigated analyte, and respond positively to the method. In general, it is the total fraction of the number of positive results assigned correctly with the method used.
- **Analytical specificity:** proportion of samples that do not contain the microorganism under investigation, and respond negatively to the method. It is the total fraction of the number of negative results assigned correctly with the method used. Analytical specificity refers to the ability of an assay to detect only the intended target, and that the quantification of the target should not be affected by cross-reactivity of other related or potentially interfering analytes.
- **Efficiency:** it is the total fraction of the number of results assigned correctly with the method used.

		Method to validate OJABIO AcN SARS-CoV-2 Rapid Test		
		(+)	(-)	Total
Reference Method ELISA	(+)	a	b	a + b
	(-)	c	d	d + d
	Total	a + c	b + d	n = a + b + c + d

The calculations to evaluate the validation data are performed as follows:

Sensitivity = $\frac{\text{True Positives}}{(\text{True Positives} + \text{false Negatives})} \times 100 = \frac{a}{a+b} \times 100$

Specificity = $\frac{\text{True Negatives}}{(\text{True Negatives} + \text{false Positives})} \times 100 = \frac{d}{c+d} \times 100$

Efficiency = $\frac{\text{True Positives} + \text{True Negatives}}{(\text{total nº of samples})} \times 100 = \frac{a+d}{n\ total} \times 100$

Note: The evaluation of False Positives and False Negatives is not included in this validation study, since the number of samples used may not be sufficient to provide a robust calculation of these parameters.

8. VALIDATION RESULTS

Annex 1 shows the primary data obtained in the validation, which begins on 07/05/2021 and ends on 07/12/2021.

Concordance evaluation

Analyzing all the results globally (117 results), the percentage of results that are concordant between the reference methods (ELISA) and the OJABIO AcN SARS-CoV-2 Rapid Test is calculated, as shown in the following table:

Results	Nº of Data (%)
**Concordance**	116 (99,15)
**No Concordance**	1 (0.85)
**Total**	100

Concordance evaluation

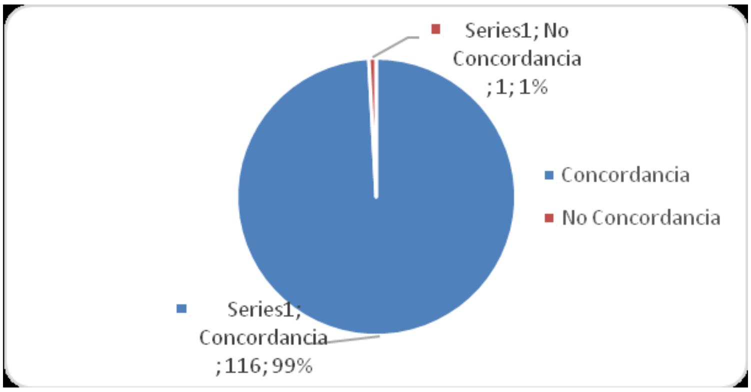

It is observed that **99%** of the results are concordant between the compared techniques.

The non-concordant results (1 of 117) correspond to a participant affected by severe Crohn's disease under treatment with anti-TNFα, which when processed by ELISA is Positive, and by the AcN SARS-CoV-2 Rapid Test of OJABIO is Negative.

Sensitivity, Specificity and Overall Efficiency Assessment

The following tables indicate the number of results obtained, associated with each of the analytical processes carried out, depending on their positivity or negativity:

		**Method to validate** OJABIO AcN SARS-CoV-2 Rapid Test		
		(+)	(-)	Total
**Reference Method** ELISA	(+)	77	1	78
	(-)	0	39	39
	Total	77	40	117

Based on the data indicated in this second table, the validation parameters are calculated:

- SENSITIVITY = **98.72 %**
- SPECIFICITY = **100.00 %**
- EFFICIENCY = **99.15 %**

9. VALIDATION CONCLUSIONS

The **OJABIO SARS-CoV-2 Neutralizing Antibody Detection Kit** is suitable for detecting neutralizing antibodies in serum samples in patients vaccinated with the two doses of the Pfizer-BioNTech vaccine against the SARS-CoV-2 coronavirus. .

Sensitivity, Specificity and Efficiency results are considered satisfactory, standing at levels of approximately **99%** or higher.

The results in which there is no concordance between the compared techniques correspond to a participant with immunosuppressive treatment.

In no case have samples been obtained that have been negative by the reference methods (ELISA) and positive by the OJABIO AcN SARS-CoV-2 Rapid Test.

After the results of the sensitivity, specificity and efficiency obtained, the **OJABIO SARS-CoV-2 Neutralizing Antibody Detection Kit** is considered technically validated for the qualitative detection of neutralizing antibodies.

Responsible for Realization

Celia García

Firmado digitalmente por Celia García
Ubicación: ALICANTE DNI: 48781020K
Fecha: 2021.07.23 23:13:11 +02'00'

Celia García
Resident
General University Hospital of Alicante

Responsible for Realization

Firmado por PARRA GRANDE MONICA - 48626653F el día 23/07/2021 con un certificado emitido por AC FNMT Usuarios

Dr. **Mónica Parra**
Head of Serology Section
General University Hospital of Alicante

Responsible for Approval

Jose Tuells

Firmado digitalmente por Jose Tuells
Fecha: 2021.07.23 03:36:01 +02'00'

Dr. **Jose V. Tuells**
Dpt. Preventive Medicine and Public Health
University of Alicante

Responsible for Approval

Firmado por JUAN CARLOS RODRIGUEZ DIAZ - NIF:05267127N el día 23/07/2021 con

Dr. **Juan Carlos Rodríguez**
Head of Microbiology Service
General University Hospital of Alicante

Annex 1. Primary data validation of AcN SARS-CoV-2 Rapid Test from OJABIO

Sample	Code	Euroimmun NeutraLISA SARS-CoV2 Assay	OJABIO AcN SARS-CoV-2 Rapid Test
1	UA02-0001	78.25	Positive
2	UA02-0002	96.93	Positive
3	UA02-0003	87.70	Positive
4	UA02-0004	94.38	Positive
5	UA02-0005	85.99	Positive
6	UA02-0006	91.32	Positive
7	UA02-0007	67.34	Positive
8	UA02-0008	99.89	Positive
9	UA02-0009	90.32	Positive
10	UA02-0010	99.28	Positive
11	UA02-0011	94.99	Positive
12	UA02-0012	72.41	Positive
13	UA02-0013	89.76	Positive
14	UA02-0014	95.38	Positive
15	UA02-0015	96.16	Positive
16	UA02-0016	95.94	Positive
17	UA02-0017	79.97	Positive
18	UA02-0018	88.87	Positive
19	UA02-0019	88.76	Positive
20	UA02-0020	70.57	Positive
21	UA02-0021	90.99	Positive
22	UA02-0022	89.65	Positive
23	UA02-0023	98.11	Positive
24	UA02-0024	77.80	Positive
25	UA02-0025	88.15	Positive
26	UA02-0026	86.04	Positive
27	UA02-0027	83.37	Positive
28	UA02-0028	98.61	Positive
29	UA02-0029	65.90	Positive
30	UA02-0030	71.74	Positive
31	UA02-0031	80.75	Positive
32	UA02-0032	72.07	Positive
33	UA02-0033	90.65	Positive
34	UA02-0034	86.82	Positive
35	UA02-0035	57.50	Positive
36	UA02-0036	58.89	Positive
37	UA02-0037	77.47	Positive
38	UA02-0038	98.11	Positive

39	UA02-0039	96.38	Positive
40	UA02-0040	66.51	Positive
41	UA02-0041	77.80	Positive
42	UA02-0042	47.26	Negative
43	UA02-0043	78.30	Positive
44	UA02-0044	83.70	Positive
45	UA02-0045	95.22	Positive
46	UA02-0046	54.94	Positive
47	UA02-0047	92.77	Positive
48	UA02-0048	99.61	Positive
49	UA02-0049	90.32	Positive
50	UA02-0050	75.13	Positive
51	UA02-0051	62.73	Positive
52	UA02-0052	86.48	Positive
53	UA02-0053	99.33	Positive
54	UA02-0054	86.93	Positive
55	UA02-0055	91.54	Positive
56	UA02-0056	94.60	Positive
57	UA02-0057	81.08	Positive
58	UA02-0058	78.25	Positive
59	UA02-0059	86.26	Positive
60	UA02-0060	98.94	Positive
61	UA02-0061	92.60	Positive
62	UA02-0062	70.13	Positive
63	UA02-0063	76.19	Positive
64	UA02-0064	94.05	Positive
65	UA02-0065	99.50	Positive
66	UA02-0066	87.59	Positive
67	UA02-0067	98.78	Positive
68	UA02-0068	71.40	Positive
69	UA02-0069	84.14	Positive
70	UA02-0070	92.16	Positive
71	UA02-0071	69.62	Positive
72	UA02-0072	51.77	Positive
73	UA02-0073	82.36	Positive
74	UA02-0074	97.44	Positive
75	UA02-0075	87.59	Positive
76	UA02-0076	83.25	Positive
77	UA02-0077	96.72	Positive
78	UA02-0078	99.05	Positive
79	16sp0000112	0.00	Negative
80	15SP0000068	0.00	Negative
81	15SP0000070	0.00	Negative

82	15SP0000071	1.13	Negative
83	15SP0000084	7.96	Negative
84	15SP0000085	0.33	Negative
85	15SP0000086	0.00	Negative
86	15SP0000087	0.00	Negative
87	15SP0000088	0.00	Negative
88	15SP0000110	0.00	Negative
89	15SP0000774	0.00	Negative
90	15SP0000786	3.72	Negative
91	15SP0000116	6.27	Negative
92	15SP0000124	1.27	Negative
93	15SP0000237	0.00	Negative
94	15SP0000288	2.17	Negative
95	15SP0000265	0.00	Negative
96	15SP0000371	0.00	Negative
97	15SP0000392	3.49	Negative
98	15SP0000403	5.33	Negative
99	15SP0000455	6.31	Negative
100	15SP0000004	3.06	Negative
101	15SP0000023	1.60	Negative
102	15SP0000056	0.90	Negative
103	15SP0000196	1.46	Negative
104	15SP0000310	4.24	Negative
105	15SP0000501	4.48	Negative
106	15SP0000528	9.38	Negative
107	15SP0000532	13.67	Negative
108	15SP0000556	8.53	Negative
109	15SP0000592	5.23	Negative
110	15SP0000606	6.46	Negative
112	15SP0000627	5.47	Negative
113	15SP0000682	5.66	Negative
114	15SP0000710	4.71	Negative
115	15SP0000716	11.59	Negative
116	15SP0000804	13.81	Negative
117	15SP0000824	12.11	Negative
118	15SP0000925	7.54	Negative
